# Physical activity as a promising alternative for young people with juvenile idiopathic arthritis: Towards an evidence-based prescription

**DOI:** 10.3389/fimmu.2023.1119930

**Published:** 2023-02-13

**Authors:** Emmanuelle Rochette, Oussama Saidi, Étienne Merlin, Pascale Duché

**Affiliations:** ^1^ Department of Pediatrics, Clermont-Ferrand University Hospital, Clermont-Ferrand, France; ^2^ Clermont Auvergne University, INSERM, CIC 1405, CRECHE unit, Clermont-Ferrand, France; ^3^ Toulon University, Laboratory “Impact of Physical Activity on Health” (IAPS), Toulon, France

**Keywords:** exercise, inflammation, metabolism, sleep, rhythms

## Abstract

Juvenile idiopathic arthritis (JIA) is the most common rheumatic disease in young people. Although biologics now enable most children and adolescents with JIA to enjoy clinical remission, patients present lower physical activity and spend more time in sedentary behavior than their healthy counterparts. This impairment probably results from a physical deconditioning spiral initiated by joint pain, sustained by apprehension on the part of both the child and the child’s parents, and entrenched by lowered physical capacities. This in turn may exacerbate disease activity and lead to unfavorable health outcomes including increased risks of metabolic and mental comorbidities. Over the past few decades, there has been growing interest in the health benefits of increased overall physical activity as well as exercise interventions in young people with JIA. However, we are still far from evidence-based physical activity and / or exercise prescription for this population. In this review, we give an overview of the available data supporting physical activity and / or exercise as a behavioral, non-pharmacological alternative to attenuate inflammation while also improving metabolism, disease symptoms, poor sleep, synchronization of circadian rhythms, mental health, and quality of life in JIA. Finally, we discuss clinical implications, identify gaps in knowledge, and outline a future research agenda.

## 1 Introduction

Juvenile idiopathic arthritis (JIA) is the most common pediatric inflammatory disease. It results from an autoimmune attack on the synovial membrane, and produces mild to severe systemic inflammation detectable by routine blood tests. Overall, the disruption of the immune system results from an overproduction of pro-inflammatory cytokines, principally tumor necrosis factor (TNF)-α, interleukin (IL)-1, and IL-6. This overproduction leads to a cascade of events at different levels: molecular, cellular, and systemic (1). Children with JIA show increased levels of autoreactive CD4+ T cells, including T helper (Th) 1 and Th17 cells, producing interferon-gamma (IFN-γ) and IL-17, respectively. This results in the production of an array of pro-inflammatory cytokines and pro-inflammatory S100 family proteins, also called calprotectin (2, 3). In parallel, the inhibition of regulatory T cells (Tregs) with decreased anti-inflammatory cytokine IL-10 results in loss of immune tolerance. An imbalance between auto-reactive Th1/Th17 and Tregs leads to the failure of T-cell tolerance to self-antigens, which contributes to both local and systemic inflammation (2, 4, 5).

In an earlier systematic review of case-control studies, we addressed physical activity and sedentary behaviors in JIA (6). We underline here that physical activity is defined as any bodily movement produced by skeletal muscles that results in energy expenditure. In daily life physical activity, can be categorized into occupational, sports, conditioning or any other activities. On the other hand, exercise is a subset of physical activity that is planned, structured, and repetitive (7). As expected, we found that children and adolescents with JIA spent less time in physical activity, especially of moderate to vigorous intensity (MVPA), than their healthy counterparts, and spent more time in sedentary behavior. Less physical activity and increased sedentary could partly be explained by disease symptoms (e.g., persistent joint pain and stiffness). However, it has been reported that the kinesiophobia associated with movement can be an even stronger deterrent than the pain itself (8, 9). JIA may thus produce a physical deconditioning spiral initiated by joint pain, sustained by apprehension on the part of both the child and the child’s parents, and entrenched by decreased physical activity and capacities. The disease and its treatments may also have a direct impact on muscle structure and/or function and on physiological adaptation during exercise. JIA not only affects joints, but also causes many other disturbances such as impaired metabolism, poor quality of sleep, depressed mood, fatigue, etc. liable to adversely affect autonomy, social life, and development. It has been reported that young people with JIA experience more difficulties managing their emotions, peer relationships, and schooling, resulting in a health-related lowered quality of life than their healthy counterparts, especially during the early stage of the disease (10–12).

Exercise is recognized as a non-pharmaceutical mode of intervention causing physiological adaptations of the immune system through myokine secretion by active muscles. These effects are mediated by a wide range of factors, including exercise-induced release of anti-inflammatory cytokines and stress hormones, and hemodynamic effects resulting in cellular reorganization (13). Exercise is also likely to act positively on metabolism, disease symptoms, sleep disturbances, the synchronization of circadian rhythms, mental health, and quality of life. Here we present an overview of the current literature supporting exercise as a behavioral, non-pharmacological alternative for JIA treatment. We then discuss clinical implications, identify gaps in knowledge, and outline a future research agenda in pursuit of evidence-based exercise prescription in JIA.

## 2 Search strategy

A MedLine search was conducted, from February 2022 to September 2022, according to published guidance on narrative reviews (14) using the following terms: “juvenile idiopathic arthritis[MeSH Terms]” OR “arthritis[MeSH Terms]” OR “rheumatoid arthritis[MeSH Terms]” associated to following terms in PubMed research: circadian clock[MeSH Terms]; Circadian Rhythm[MeSH Terms]; Period Circadian Proteins Sleep Disorders[MeSH Terms]; Circadian Rhythm[MeSH Terms]; Sleep[MeSH Terms]; metabolism[MeSH Terms]; lipid metabolism[MeSH Terms]; carbohydrate metabolism[MeSH Terms]; exercise[MeSH Terms]; Sedentary Behavior[MeSH Terms]; Mental Health[MeSH Terms]; quality of life[MeSH Terms]; physical fitness[MeSH Terms]; cardiorespiratory fitness[MeSH Terms].

## 3 Effect of exercise on inflammation

Exercise-induced inflammation was documented in early sports science studies, where a notable elevation in circulating IL-6 was reported (15). However, a temporal variation in cytokine levels following exercise was also documented (16). Briefly, immediately after exercise, IL-6 is the first cytokine secreted in response to muscle contraction (17–19). Muscle contraction leads to an increase in cytosolic Ca2+ and activation of the p38 MAPK pathway and calcineurin, which leads to the activation of upstream transcription factors and the secretion of IL-6 (20, 21). This pathway results in an anti-inflammatory effect of IL-6, which contrasts with the NFκB-induced production of IL-6 and other cytokines such as IL-1β or TNF-α by macrophages (20). In response to exercise, IL-6 (principally of muscular origin) therefore induces an anti-inflammatory environment within the next few hours *via* the production of IL-1ra and IL-10. It also inhibits the production of TNF-α (18, 22).

IL-6 acts through a heterodimeric signaling complex consisting of the IL-6 receptor (IL-6R) and the signal-transducing subunit glycoprotein 130 (gp130). IL-6R occurs in both soluble (sIL-6R) and membrane-bound forms (mIL-6R), distinguishing pro-inflammatory IL-6 trans-signaling (via the sIL-6R) from anti-inflammatory IL-6 classic signaling (via mIL-6R) (23). One study suggested that sIL-6R concentration after exercise reflected changes in leukocyte subpopulations migrating into damaged tissues, with an initial post-exercise period that reflects signaling for massive monocyte/macrophage infiltration and a later stage of post-exercise recovery (the regenerative phase) marked by low levels of sIL-6R (24). There is also abundant evidence that IL-6 in combination with sIL-6R plays a role in nociception and inflammatory hyperalgesia (25, 26).

In response to acute exercise, plasma IL-6 levels increase nonlinearly over time, peaking at the end of exercise and then falling to the pre-exercise level (27). By contrast, there is a negative association between basal plasma IL-6 levels and amount of regular physical activity, low basal plasma IL-6 correlating to higher physical activity level (27). Regular physical activity entails multiple adaptations including increased pre-exercise skeletal muscle glycogen content, increased oxidation of intramuscular triglycerides, and enhanced activity of key enzymes involved in ß-oxidation (28, 29). Skeletal muscle consequently increases its capacity to oxidize fat and becomes less dependent on plasma glucose and muscle glycogen as substrates during exercise (27). For IL-6 gene transcription, pre-exercise intramuscular glycogen content is an important stimulus, and so transcription rates are higher when glycogen levels are lower (17, 18). Regular physical activity thus appears to downregulate plasma IL-6 but to upregulate the mIL-6 receptor (20, 30). In response to regular exercise, basal IL-6R mRNA content in trained skeletal muscle is increased by approximately 100%, suggesting that the downregulation of IL-6 is partially counteracted by an enhanced expression of IL-6R, whereby the sensitivity of skeletal muscle to IL-6 is increased (20, 27, 30). Finally, in healthy adults, studies on the effect of regular physical activity on inflammatory markers at rest or after an exercise bout of resistance have essentially found that training intensity plays a role in cytokine responses, higher intensity (> 70% of one maximum repetition (1RM)) eliciting a more favorable response (28).

In children with JIA, a single 20-minute exercise bout at 70% of maximal heart rate at 8:30 am induced a slight transient increase in the level of plasma calprotectin (MRP 8/14, a pro-inflammatory polypeptide), together with transient self-evaluated pain, but no significant change in IL-6 or soluble IL-6 receptors (31). However, at 24 h post-exercise, calprotectin, IL-6 and pain decreased compared to control-day levels (32). In JIA, acute exercise thus induces a dissociated physiological response over time. Unfortunately, there are still no data on the effect of long-term exercise on inflammatory markers in children with JIA. JIA, with its seven subcategories, is a complex, heterogeneous disorder, and the treatments used differ depending on the form and persistence of the disease (from the use of NSAIDs alone to biologics). It is therefore difficult to draw any simple conclusion on response and physiological adaptations to exercise in this population. Future work must therefore study the impact of acute exercise or regular physical activity on inflammatory markers according to JIA category, disease activity, and treatments. Finally, the physiological responses in this population must be evaluated according to different exercise variables: type (endurance, resistance), duration, and intensity.

## 4 Effect of physical activity on metabolism

In earlier work, we found that children with JIA presented impaired energy metabolism during exercise (deficient lipid oxidation) even when the disease was inactive, and that this impairment was less marked in children treated with TNF-α inhibitors (33, 34). These data suggest muscle dysfunction in children with active or even inactive JIA. This may result from subclinical inflammation, muscle changes secondary to physical deconditioning, or impaired muscle oxidative function. Lastly, assessment of muscle structure and function showed a reduction of intermuscular adipose tissue (IMAT) possibly linked to lipid oxidation deficiency during exercise (35).

Altered metabolic control of lipid and glucose homeostasis predispose to developing cardiovascular diseases (CVDs) such as type 2 diabetes and atherosclerosis (36). Accumulated lipids in vascular walls cause chronic inflammation, which favors the long-term advent of atherosclerosis. Children with JIA may thus be at increased risk of developing CVD in adulthood (37, 38). Better characterization of the lipid profile in this patient population, focusing in particular on sphingolipids, seems a promising avenue. Ceramides belong to the sphingolipid family and have biological actions in cell proliferation, differentiation, and death. They are also involved in the signaling pathways in play in insulin resistance, oxidative stress, and inflammation (39). Inflammatory cytokines, in particular TNF-α, are correlated with several plasma subspecies of ceramides, notably ceramide C24:0 (40). Finally, disturbed intracellular sphingolipid metabolism has been implicated in the onset of several diseases such as obesity, type 2 diabetes, atherosclerosis, and cardiovascular disease, and also rheumatoid arthritis (RA) (41, 42). We must therefore endeavor to restore optimal metabolism in children with JIA.

Skeletal muscle is an insulin-sensitive organ responsible for 85% of glucose uptake in humans (43). In skeletal muscle, glucose homeostasis is dependent on insulin signaling, which mediates the various steps of glucose metabolism. Insulin signaling is a complex process: the binding of insulin to its receptor induces auto-phosphorylation and phosphorylation of tyrosine residues of IRS (insulin receptor substrate) proteins, thus initiating the intracellular signaling cascade (43, 44). IRS-1 is more closely related to glucose homeostasis, whereas IRS-2 is involved primarily in lipid metabolism (44). Inflammation and insulin resistance are closely linked, and inflammatory cytokines such as TNF-α, IL-6, IL-1 and IL-8 can inhibit insulin signaling by multiple mechanisms (45). TNF-α induces phosphorylation of IRS-1 on serine in place of tyrosine residues and promotes insulin resistance (46, 47). This phosphorylation of serine residues terminates the physiological activation of the receptor, thus stopping the insulin signal (48). TNF-α is also involved in lipid metabolism: it has been shown to promote lipid accumulation, alter mitochondrial ultrastructure and function (49), suppress AMP-activated protein kinase (AMPK) activity, and reduce fatty acid oxidation (50). Finally, it has been shown that *in vitro*, TNF-α stimulates the production of diacylglycerol (DAG) and ceramide, which are involved in the pathogenesis of insulin resistance in skeletal muscle (51). Regarding the effect of IL-6 on muscle metabolism, a distinction must be made between acute effects (in response to physical exercise) and chronic effects (chronic inflammation, obesity). In an acute context of physical exercise, the increase in IL-6 (the lower the level of muscle glycogen, the greater the increase (52)) improves insulin sensitivity and glucose uptake *via* activation of AMPK (49, 53, 54). IL-6 also promotes lipid metabolism by increasing lipolysis and fatty acid oxidation in myocytes and adipocytes, and in the whole body (49, 53, 54). In addition, *in vitro* data have shown that IL-6 induces translocation of the transporter GLUT4 to the plasma membrane of myotubes (53). By contrast, in a chronic context, IL-6 is associated with insulin resistance and inflammation in skeletal muscle and liver (49).

Inflammatory diseases such as juvenile idiopathic arthritis are treated, for the most severe forms, using biotherapies. These treatments target certain pro-inflammatory cytokines including TNF-α (etanercept, adalimumab, infliximab). In adults with rheumatoid arthritis, several studies have shown that anti-TNF treatment increases insulin sensitivity (44, 55–58). Anti-TNF antibodies restore the phosphorylation state of Ser312-IRS-1 and AKT, important mediators of the insulin signaling cascade (44). On the other hand, in children with JIA, we found only one study on the effect of anti-TNF-α on glucose metabolism. It reports no difference in plasma glucose levels before and after 3 and 6 months of treatment with anti-TNF-α (59). Regarding the effect of anti-TNF-α on circulating lipid levels, in children with JIA, studies report that after 12 months of treatment with etanercept, the triglyceride level significantly decreased (60, 61).

Lipid oxidation capacity is related to physical condition, itself related to level of physical activity. Impaired lipid metabolism may therefore be the whole body’s adaptation to a state of low activity and correspondingly low energy expenditure in children with JIA (62, 63). In this case, the system could be remobilized by increasing physical activity and incorporating a regular physical activity program. Regular exercise is associated with an increase in the proportion of oxidized lipids during the exercise and an improvement of mitochondrial enzymatic capacities, especially those involved in lipid β-oxidation (64). Physical exercise also improves glucose transport and increases the expression or activity of entities involved in insulin-signaling pathways, such as protein B kinase (Akt) and AMPK, leading to an increased insulin sensitivity (65). Prescription of regular physical activity could thus help correct this energy metabolism disturbance. Implementing exercise training programs in children with JIA should therefore improve their lipid metabolism and reduce the risk of CVD developing during their adulthood.

## 5 Effect of exercise on circadian rhythms

**Figure 1:**
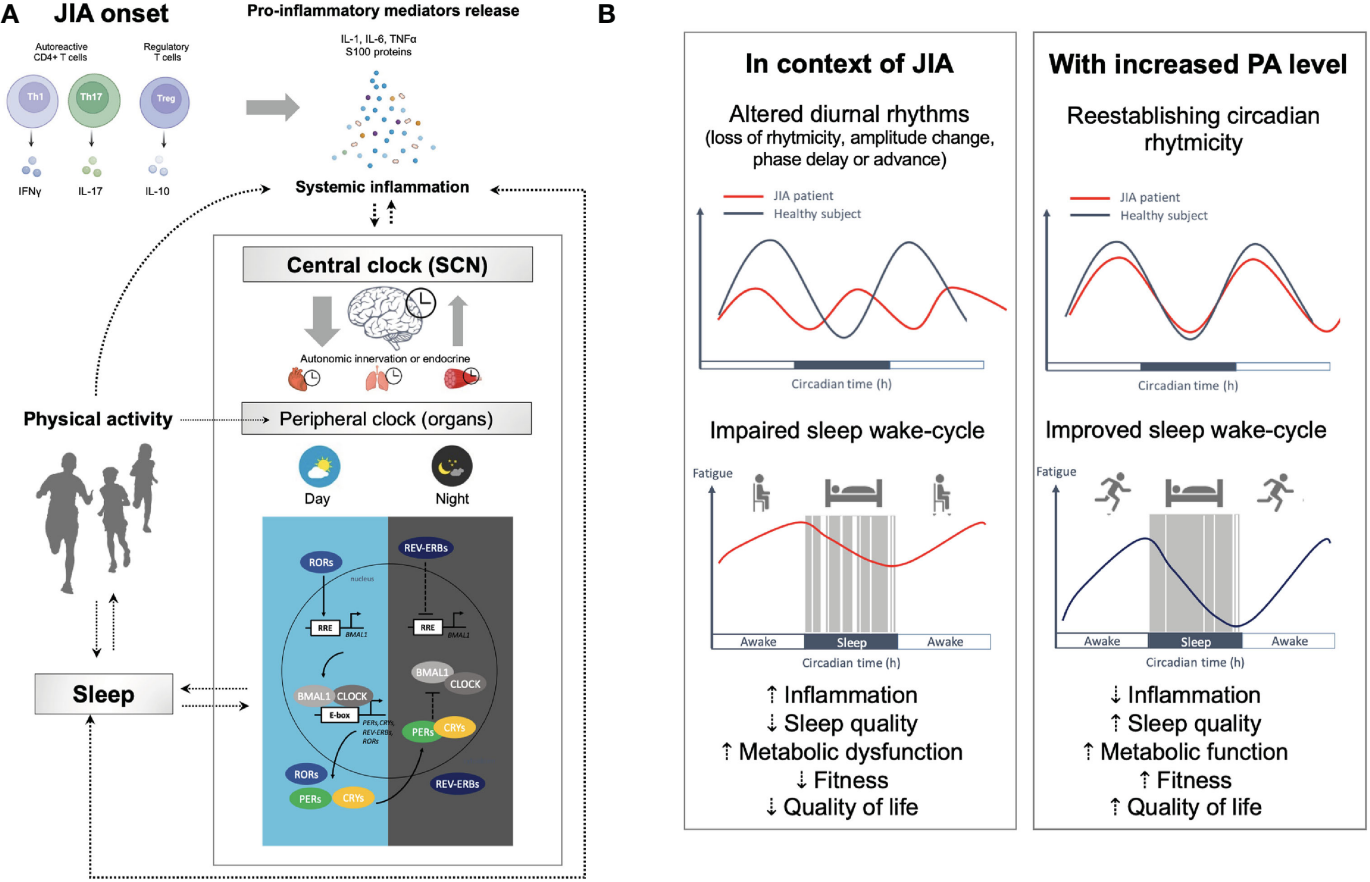
The circadian clock system, inflammation, and multiple disorders in JIA. **(A)** Under physiological conditions, the expression levels of the clock genes undergo circadian oscillation in the central (suprachiasmatic nucleus [SCN]) and peripheral clocks (e.g., brain, muscle, immune cells, etc.), mediated by transcriptional and translational feedback loops between the CLOCK/BMAL1 transcriptional activator complex and its repressors (PER/CRY, REV-ERBα) or activators (RORα/β) to drives the expression of multiple clock-controlled genes. A healthy intact clock is necessary for body homeostasis. Desynchronization between the cellular oscillators in the central clock and those in peripheral clocks results in circadian disruption. Clock misalignments are therefore detrimental to fitness and perturb metabolic function and the immune system, which can result in chronic health disorders. The disruption of oscillatory rhythms of clock genes thus perturbs functions of metabolism-related genes controlled by the molecular clock, and dysregulation of circadian rhythms are involved in the initiation and progress of rheumatic diseases, leading to a vicious cycle of inflammation. **(B)** Yet the involvement of circadian rhythm remains barely explored in JIA, we hypothesize that behavioral, endocrine and immune circadian rhythmicity could be altered in young people with JIA. Possible changes to rhythmicity include loss or gain of rhythmicity, dampening or increase in the amplitude, phase advancement or delay, and base shift. In JIA, multiple disorders have been described that involve not just joints but many other organs (impaired metabolism, poor quality of sleep, depressed mood, fatigue, etc.). Physical and mental fatigue are both reinforce by CNS inflammation mediated by pro-inflammatory cytokines (e.g., TNF-α; IL-1; and IL-6) that affects monoamine neurotransmitter systems resulting in altered cognition, mood and motivation (74–77). The circadian system allows for adequate partitioning of physiology corresponding with environmental signals coordinating metabolism, immunity, activity, and feeding behavior with the organism’s environment. Environmental changes (e.g., changes in light exposure and exercise) can affect the rhythmicity of gene expression under circadian clock control. Overall, there is evidence that exercise is a potential time cue to synchronize rhythms and that it could maximize therapeutic benefits at specific phases in JIA. Physical activity, through a wide range of factors, including exercise-induced release of anti-inflammatory cytokines, stress hormones, and hemodynamic effects resulting in cellular reorganization, is known to impact the mechanisms involved in JIA associated disorders. Holistic coordination of circadian rhythms (sleep-wake cycle, meal timing, and social habits), the therapy schedule, and exercise could improve sleep quality and alleviate symptoms in patients with JIA. BMAL1, Brain and muscle arnt-like 1; CLOCK, Circadian locomotor output cycles kaput; CRYs, Cryptochromes; PERs, Periods; REV-ERVs. Nuclear receptors encoded by nuclear receptor subfamily 1, group D (NR1D); RORs, Retinoic acid receptor-related orphan receptors; RRE, REV-ERB/ROR responsive element.

Physiological functions follow circadian rhythm under the control of the circadian system (66). Transcription factors CLOCK (circadian locomotor output cycles kaput) and BMAL1 (brain and muscle aryl hydrocarbon receptor nuclear translocator-like protein 1) are major regulators of peripheral clock gene expression (67). These proteins reach their peak activity during the inactive phase (i.e., at night) and bind to the enhancer (E)-box elements in the nucleus to drive the transcription of the Period (PER) and Cryptochrome (CRY) genes, which exhibit peak activity at the beginning of the active phase and inhibit CLOCK and BMAL1 activities. The opposite effects on the feedback loop are driven by the retinoic acid-related orphan receptors (ROR), which activate REV-ERBs (receptor subfamily 1 group D member 1) which inhibit BMAL1 transcription (68). Some of these clock proteins have central functions in inflammation and metabolism such as REV-ERBα regulates skeletal muscle oxidative capacity by modulating mitochondrial biogenesis and fatty acid oxidation (68). It reduces the production and release of IL-6 and inhibits the expression of Th17-mediated pro-inflammatory cytokines; its overexpression in turn inhibits the development of Th17 cells (69). Through activation of the nuclear factor κB (NF-κB), clock genes are implicated in inflammatory response, with activation of pro-inflammatory cytokines by CLOCK and an anti-inflammatory role *via* BMAL1 (70). However, under chronic systemic inflammation, IL-1β or TNF-α may inhibit the E-box-dependent gene expression mediated by the BMAL1/CLOCK complex (67). The disruption of oscillatory rhythms of clock genes thus perturbs functions of metabolism-related genes controlled by the molecular clock (67), and dysregulation of circadian rhythms are involved in the initiation and progress of rheumatic diseases (71). On other hand, autoimmune and inflammatory diseases can directly affect clock gene expression, leading to a vicious cycle of inflammation and detrimental effects on treatment response (69, 72, 73). Yet the involvement of circadian rhythm remains barely explored in JIA. Given that JIA shares clinical and pathological features with rheumatoid arthritis (RA), some findings from RA studies are also mentioned by analogy to support some of the hypotheses advanced in what follows (**Figure 1**). However, we emphasize that further evidence must be sought in future studies of young people with JIA.

Recent studies report a preserved behavioral, endocrine and immune circadian rhythmicity in RA but a greater time-of-day variation in gene expression profile, with 104 genes differing between morning and afternoon against only 25 in healthy controls (78). Interestingly, the studies have shown an enrichment at dawn for transcription factor binding motifs including STAT3, an important signaling mediator of IL-6 action. Finally, they also report a rhythmic increase in serum lipid changes particularly in the ceramide class. Acrophase of the rhythmic ceramides occurred only in RA at 11:00 pm and most of the rhythmic lipids peaked at 6:00 am or 6:00 pm in both healthy controls and RA patients. The authors therefore hypothesize that chronic joint inflammation serves as circadian organizer, coupling lipid-metabolic pathways to the core clock (78). The same team has shown in a mouse model of RA that chronic inflammatory arthritis drives major changes in muscle and liver energy metabolism, revealing alterations in lipid metabolism and mitochondrial function. With impaired β-oxidation and sphingolipid and ceramide accumulation most pronounced during the day, this demonstrates that rhythmic inflammation of joints drives a time-of-day-dependent build-up of bioactive lipid species (79). Although many findings in RA need to be verified in JIA, these data still remind us that metabolism and inflammation are under the control of the circadian clock and that rheumatic diseases show specific rhythmicity, which may also be the case in JIA. Symptoms of JIA, like those of RA, follow circadian rhythms, with an increase in activity in the early morning, a reduction during the day, and then a smaller increase in the early evening. Some studies have reported a temporal relationship between elevated levels of pro-inflammatory cytokines and symptoms in arthritis, such as morning stiffness (80, 81). Pro-inflammatory cytokines show a specific rhythmicity that differs from that observed in healthy controls. The IFN-γ/IL-10 ratio peaks at 4:00 am (synchronous with the cortisol nadir) and troughs at 3:00 pm. There is therefore a predominance of cellular immunity during the night and early in the morning when the IFN-γ/IL-10 ratio is high (5, 82). These data are consistent with the observation that joint symptoms in both JIA and RA are more severe at night and early in the morning. In healthy subjects, TNF-α peaks at 3:00 am and IL-6 peaks at 6:00 am, whereas in patients with RA, the peak of TNF-α is later at 6:00 am and that of IL-6 later at 7:00 am (80). By contrast, although we have observed a well-marked at-rest peak in IL-6 and calprotectin at 3:00 pm in children with JIA (31), data on cytokine circadian rhythm disruption in these disorders are still lacking.

Exercise is a robust zeitgeber of skeletal muscle clocks. It can reset the molecular circadian clock and effectively improve the negative effects of disrupted sleep patterns on circadian rhythm (83). The phase-shifting effects of exercise on mammalian circadian rhythms are thought to be mediated in part by serotonin, neuropeptide Y and melatonin, leading to acute changes in PER1 and PER2 expression (84). Also, several studies have found that exercise can modulate circadian rhythms and time-cue peripheral tissues. In the middle of the active phase, with increased contractile activity and higher metabolic demands, most circadian muscle genes show peak expression. In human skeletal muscle, resistance exercise (10 sets of eight repetitions of isotonic knee extension at 80% of the predetermined one-repetition maximum) can therefore directly regulate the circadian clock genes *Per2, Cry1* and *Bmal1* (84). For example, BMAL1 expression increase 1.6-fold 4 h after exercise, and 3.5-fold 8 h after an acute aerobic exercise bout of 70 min at 70% of the VO_2max_ in trained adults (85). On the other hand, exercise capacity can be modulated by the expression of BMAL1, CRY1 and PER2, ***via*** the regulation of mitochondrial function and the modulation of lipid and glucose metabolism (68). Of note, Basti et al. (86) report that circadian oscillation of exercise performance seemed dependent in amplitude on BAML1 expression and in phase on expression of PER2 (86). Finally, in healthy adults, an acute bout of exercise (maximal progressive exercise on a cycle ergometer) modified expression patterns of CLOCK and BMAL1 in CD4+ T cells and cytokine production in a training status-dependent manner (70). Subjects that participate in regular physical activity presented after the same acute bout of exercise an improvement in anti-inflammatory profile, with higher levels of IL-10 and augmented expression of CRY1 and REV-ERBα, with positive correlation between clock genes expression in CD4+ T cells and physiological parameters (VO_2max_ and power) (70). A growing body of evidence points to circadian clock genes, particularly *RORs* and *REV-ERBs*, as promising therapeutic targets in autoimmune diseases (69). Given that exercise induces increased expression of REV-ERBs and that REV-ERBα reduces the production and release of IL-6 and inhibits the expression of Th17-mediated pro-inflammatory cytokines (69), exercise is potentially helpful for managing JIA.

Importantly, in typically developing children, maturational changes lead to a circadian phase delay arising from the circadian timing system during adolescence. The transition from childhood to adolescence brings a delay of roughly 2 h in dim-light melatonin onset (DLMO) (87). This delay might be affected by JIA pathogenesis, but no study has yet explored this hypothesis. However, some studies have revealed abnormalities in melatonin secretion in both JIA and RA (88, 89). The disease activity scores in JIA and erythrocyte sedimentation rate (ESR) were positively correlated with serum melatonin levels (88). Here we underline that morning exercise can mediate a phase shift in rhythms and was effective in bringing forward the circadian phase in healthy adolescence (90, 91). However, how far these results can be transposed to young people with JIA needs to be addressed in future matched case-control studies.

## 6 Effect of physical activity on sleep disturbances

We recently made a comprehensive systematic review and meta-analysis of case-control studies on sleep in JIA. Despite an unchanged sleep duration, we found evidence that young people with JIA found more difficulty initiating and maintaining sleep than their healthy counterparts (92). More importantly, poor sleep quality was found to potentiate functional disabilities, and to increase fatigue and excessive daytime sleepiness. The biological mechanisms behind sleep disturbances in young people with JIA are varied and complex. Putative contributory factors include chronic subclinical inflammation, the disease symptoms (e.g., pain and fatigue), and associated sleep disorders. These detrimental effects could be implicated in part in the excessive inflammation, and also in secondary phenomena such as metabolic disturbances and endocrine dysregulation (93).

Exercise is known to have a beneficial effect on sleep, increasing both its duration and its quality. There is an extensive literature on this topic in adults (94, 95). Meta-analyses conducted in the 1990s concluded that exercise had a positive effect on sleep, albeit small (96, 97). However, we note that many of the studies included in these meta-analyses were conducted in good sleepers, thus limiting the potential improvement of sleep through a ceiling effect. The meta-analysis of Kredlow et al. (95), including 66 studies, 45 of which tested the effect of acute exercise on sleep, finds that acute exercise has a small beneficial effect on total sleep duration, time to sleep, sleep efficiency, stage 1 sleep, slow-wave sleep (SWS), and rapid eye movement (REM) sleep, along with a moderate decrease in wake after sleep onset. On the other hand, chronic exercise exerts small-to-moderate beneficial effects on several sleep outcomes. In particular, chronic exercise seems to improve sleep initiation and continuity. Although the positive effect of exercise on sleep is accepted, characteristics such as intensity, energy expenditure, modality (endurance, strength), and timing can all play crucial roles in modulating sleep physiology (95). Other individual characteristics such as age, physical condition, chronotype, and sleep quality at baseline may interfere with observed effects (95).

An early study by Dworak et al. (98) showed that high-intensity acute exercise resulted in higher sleep efficiency, decreased sleep latency, a higher proportion of SWS, and less time spent in stage 2 in healthy adolescents (98). Lang et al. (99) confirmed a positive effect of overall physical activity on sleep during adolescence. The effect of physical activity on sleep in adolescents remained significant even after controlling for confounding factors such as psychological functioning (99). Even so, the authors suggest that the strength of this relationship is underestimated due to biases related to the limited reliability and validity of assessment methods. Recent evidence has continually supported the relevance of exercise intervention on sleep in pediatric populations with chronic diseases, especially those with obesity or obstructive sleep apnea (OSA) (100–102). However, fewer studies have examined the effect of exercise on sleep in JIA. Ward et al. (103) found that patients with JIA who met the obstructive apnea hypopnea index (OAHI) clinical criteria for obstructive sleep apnea (≥ 1.5) had more sleep disturbances, higher fatigue, and lower quality of life (103). Moreover, recent studies have confirmed that young people with JIA are at increased risk of developing OSA (104). Physical activity programs may thus be useful in JIA to mitigate both sleep disturbances and obstructive sleep apnea.

## 7 Effect of physical activity on physical fitness, quality of life, and mental health

Chronic health disorders are associated with some general or weakly specific symptoms that affect general condition and help to account at least in part for a deterioration in quality of life. Among these general signs are physical deconditioning, fatigue, and pain. These signs can worsen during “flare-ups” of the disease and/or through treatment. The process of deconditioning results from the adaptation of all the body’s systems to a state of lowered activity and attendant low energy expenditure. It is characterized by a marked decline in functional abilities and general physical condition, resulting in a debilitated family and social life, and a decrease in functional independence, quality of life, and self-esteem (105, 106). We then observe an increase in fatigue and an alteration in cardiorespiratory capacity and functional muscle properties. When a chronic disorder occurs in childhood, the development of physical, metabolic, and muscular capacities can thus be disturbed by the disease and its associated treatments, and also by undernutrition, reduced physical activity, or a sedentary lifestyle. The oldest studies dating from before 2015 report not only a decrease in aerobic and anaerobic capacities (107–112) and isometric strength (105, 113–115), but also altered body composition in JIA patients, whose fat mass increased and bone and lean mass decreased (116, 117). These findings were for both active and undetected disease, but loss of fitness seemed to correlate with disease severity (110). More recent studies since biologics have become available instead show suboptimal muscle strength and bone mineral density compared to controls, but no differences in cardiorespiratory fitness or body composition (118, 119). All the literature on the impact of physical training in children with JIA report no adverse effect; results are in favor of exercise intervention with improved quality of life, aerobic capacity (VO_2_ peak), functional ability (CHAQ: Childhood Health Assessment Questionnaire), strength, bone mineral density, and range of motion (109, 120–125).

The results for pain are controversial or neutral with no effect of physical activity (126–130). Increased pain can be associated with a drop in the level of serotonin and norepinephrine, which is also linked to depression. It is thus commonly observed that patients with JIA experiencing pain and disability are also predisposed to low mood and anxiety (131). One quarter of children with JIA report moderate to severe symptoms of anxiety and depression, associated only with pain and stress (132, 133). Some evidence suggests that inflammation is implicated in the neuroimmune cascade resulting in fatigue, cognitive impairment, and depressive symptoms. JIA patients with high disease activity are characterized by higher kynurenine/tryptophan (KYN/TRP) ratios and lower TRP levels, reflecting increased activity of indoleamine-2,3-dioxygenase (IDO) (134). By contrast, patients under methotrexate (MTX) show a significant fall toward more normal values of KYN/TRP ratio (134). IDO levels are inversely correlated with serotonin levels and lowered serotonin concentrations and may result in reduced melatonin levels. This may in part contribute to the sleep disturbance observed in JIA patients. Consistent with this, sleep quality and depressive symptoms are improved in adults by infliximab treatment (135). Exercise has an antidepressant effect not only through the social benefits of group exercise, but also by improving self-esteem and self-efficacy and finally by improving the quality of sleep (136). It is accepted that during adolescence, physical activity and exercise strengthen individual brain regions and large-scale neural circuits to improve emotional and behavioral regulation (137). Moreover, at biological level in response to aerobic exercise, KYN levels in the circulation and central nervous system can be reduced (138). Furthermore, following chronic exercise, IDO activity is decreased, the content of metabolic product KYN in the TRP/KYN pathway in the brain is reduced, and the concentration of free TRP is increased, so that it can enter the brain more easily through the blood-brain barrier and be metabolized into bioactive compounds such as serotonin and melatonin, which could decrease depression severity (138).

There is a growing body of literature on the impact of physical activity on physical fitness. However, the studies are difficult to compare in terms of the population studied (JIA subtype, treatment used, and patient age) and the physical training used, which range in intensity, duration, frequency and modality. Data on the impact of physical activity on mental health and sleep quality in JIA are lacking. Further research is needed, paying special attention to the assessment of physical activity level using both objective and subjective methods, to measure the impact of physical activity on physical fitness, mental health, and sleep in patients with JIA.

## 8 Future directions: Toward an evidence-based exercise prescription

**Figure 2:**
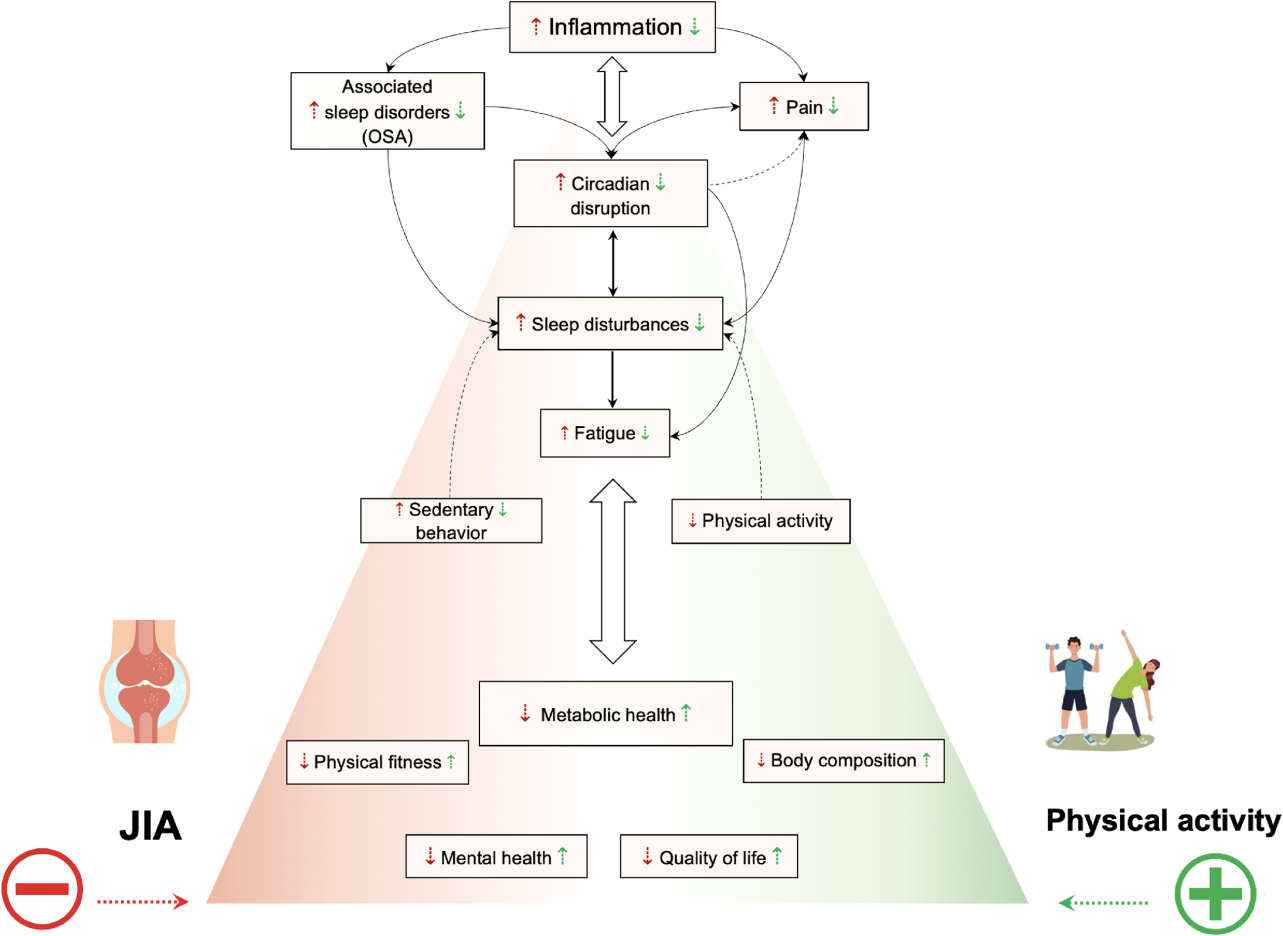
Summarized effects of exercise or physical activity on inflammation, metabolism, circadian rhythms, sleep disturbances, physical fitness, quality of life, and mental health. A vicious downward spiral emerges in children and adolescents with JIA. Immune system dysregulation leading to systemic pro-inflammatory cytokines have impact on circadian rhythms. Therefore pain and sleep are impacted. Altered sleep in turn may exacerbate inflammation, impair pain perception, and body energy restitution function which lead to diurnal fatigue, decreased physical activity level, increased sedentary and worsening disease activity and symptoms. Consequently, it results in metabolic dysfunction, impaired physical fitness, altered body composition and lower quality of life and mental health. Physical activity is likely to act positively on inflammation, metabolism, sleep disturbances, the synchronization of circadian rhythms, mental health, and quality of life. So, increase the level of physical activity and reduce sedentary behaviors in patient with JIA may be associated with holistic improvements at physiological, behavioral, or functional levels.

Overall, evidence suggests that physical activity may be associated with holistic improvements in JIA patients at physiological, behavioral, or functional levels (**Figure 2**). For practitioners, the question is therefore not whether they should recommend exercise, but rather what type or mode of exercise it should best be, and how it should be implemented. Interventions designed to increase overall physical activity level could also be a promising alternative. Above all, it is clearly advantageous to increase the level of physical activity and reduce sedentary. Physical activity includes all activities that involve body movement and are part of playing, walking, working, traveling, engaging in sports, exercise conditioning or training, and recreational activities. For better compliance and to reduce sedentary behaviors, children with JIA must also be offered physical activities compatible with their symptoms, their lifestyle, and their level of fitness. Nevertheless, it may well be that the effects of physical activity on JIA-induced inflammation in the short term are different from those obtained in the long term. Longitudinal studies of inflammatory profile evolution in response to physical activity programs are needed to draw firm conclusions.

More importantly, physical activity characteristics (intensity, duration, modality, and time of day) may differentially impact feasibility and benefits. Studies in adults that examined the time-of-day effect of exercise serum concentrations on IL-6 responses found a time-of-day adaptation, with a greater increase in concentrations of IL-6 in evening than in morning exercise (139). Elevation in IL-6 concentration persisted throughout the day after morning exercise, an outcome likely linked to the circadian rhythm of IL-6 (140). This was not found after evening exercise because there were no sampling kinetics during the night. Moreover, sleep loss is associated with next-day increase in IL-6 and TNF-α, which have been proposed as mediators of excessive diurnal sleepiness (141). In a study evaluating the effect of sleep deprivation on the cytokine response to exercise, it was shown that afternoon exercise-induced IL-6 increased during the 1 h recovery period after exercise was affected by sleep deprivation at the end of the night (141). However, we note that chronotype and time to waking are important factors that influence physiological response to exercise (142), but these data came from healthy adults and we found no data for children or JIA. The impact of the timing of exercise in the context of JIA therefore needs to be further explored. As these children may be taking disease-modifying antirheumatic drugs (DMARDs, i.e., methotrexate or TNF-α inhibitor and IL-6 inhibitor) and because pubertal status, physical training and BMI appear to be factors involved in the impact of exercise-induced IL-6 release in children (143, 144), it is necessary to test different exercise modalities (intensity, duration, type) and the impact of morning or evening practice in this population. Insofar as some studies suggest that exercise could impact both inflammation and metabolism differently according to time of practice, chronoexercise could offer a new path of action in the management of JIA.

**Table:** 

Research agenda
– For a better understanding of the effect of exercise (acute/chronic) on inflammation, future studies are needed to explore the effect of exercise on inflammatory markers according to JIA category, disease activity, and treatment. – Further studies assessing the long-term effect of JIA on metabolic alteration and comorbidities (risks of obesity, type 2 diabetes, atherosclerosis, and cardiovascular disease during adulthood) is required. Comparison between participants who meet physical activity guidelines and those who do not is necessary to assess the potential protective effect of physical activity on metabolic health in young people with JIA during adulthood. – Owing to paucity of evidence, future studies on the circadian rhythms of young people with JIA compared to their healthy counterparts are needed. Exercise may offer potential in reestablishing circadian rhythmicity in healthy individuals. However, this needs to be explored in young people with JIA. – The effect of exercise (acute/chronic) on sleep quality, fatigue, mood, and quality of life in young people with JIA needs to be researched. – Further research studying the link between inflammation sleep, circadian rhythm, physical activity and JIA symptoms and functions is needed.
Practical points
– The implementation of interventions increasing total physical activity and limiting sedentary in young people with JIA are needed. – Studies exploring the differential effect of exercise characteristics (intensity, duration, modality, and time of day) are needed to pave the way for evidence-based exercise prescription in young people with JIA. – Strategies combining exercise with other therapeutics (chronotherapy, nutrition, etc.) may potentiate the efficacy of management and care in JIA.

## 9 Conclusion

Physical activity may offer potential therapeutic benefit in children with JIA. All together evidences show that exercise is a potential time cue to synchronize rhythms and that it could maximize therapeutic benefits at specific phases. Restoration of circadian misalignment in JIA by physical activity can alleviate inflammation, improve sleep and relieve disease symptoms. So, it is clearly advantageous to increase the level of physical activity and reduce sedentary behaviors. Yet for better compliance, children with JIA must be offered physical activities compatible with their symptoms, their lifestyle, and their level of fitness. However, to date the best physical activity modalities and timing in the treatment schedule to maximize benefits for quality of life, pain, fatigue, sleep, and inflammation are yet to be determined.

## Author contributions

The authors’ responsibilities were as follows: ER, OS, ÉM, and PD conceived the review. ER and OS drafted the manuscript. ÉM and PD critically reviewed the manuscript. All authors contributed to the article and approved the submitted version.
